# Supplementary material for: Assessing afebrile malaria and bed-net use in a high-burden region of India: Findings from multiple rounds of mass screening
Source: PLoS One. 2023 Jul 19;18(7):e0287667. doi: 10.1371/journal.pone.0287667 (PMC10355393; doi:10.1371/journal.pone.0287667)
Supplement: Attachment — Submitted filename: Response to reviewers.docx [file pone.0287667.s003.docx]

**Response to reviewer comments:**

Reviewer #1: Dear Authors,
This reviewer is happy with the answers given to the scientific questions raised at the first revision.

However, as suggested by the editorial during the first revision, I think that the manuscript would be benefited of a thorough scientific and English language revision. There are many sentences difficult to read and the text does not flow well at all. It happens with some verb structures and there is an abuse of phrasal verbs. One example is the use of the verb "tally", it is not clear what you mean by this, it has the meaning of "counting" or the meaning of "recording"? Changing some sentences to a more scientific standard language will help your manuscript to flow in a better way.
I suggest to the authors to check similar manuscripts that have been published in this journal:
- <https://journals.plos.org/plosone/article?id=10.1371/journal.pone.0232874>
- <https://journals.plos.org/plosone/article?id=10.1371/journal.pone.0210578>
- <https://journals.plos.org/plosone/article?id=10.1371/journal.pone.0282209>

Other suggestion that you may like to take into account is to add at least one figure to the manuscript, I understand the issue with the copyright of Figure 1. Maybe you would like to built a similar figure by your own so we can understand the geographical setting of the study, or maybe some of the main data can be shown in a barr diagram or something similar.

**Response**: We thank the reviewer for the valuable suggestions.

We have reviewed and edited the manuscript to improve language and flow. We have got the language reviewed by a senior researcher.

We have removed the verb “tally” and edited the sentence to improve clarity.

We have created the map to show the geographical setting of the study using an open-source application (MAPWINDOW) and edited the base maps available from that source. We have added the reference to this software.

We have included a chart to report the data on malaria positivity and share of afebrile malaria in different rounds of screening.
