# Supplementary material for: Assessing afebrile malaria and bed-net use in a high-burden region of India: Findings from multiple rounds of mass screening
Source: PLoS One. 2023 Jul 19;18(7):e0287667. doi: 10.1371/journal.pone.0287667 (PMC10355393; doi:10.1371/journal.pone.0287667)
Supplement: Attachment — Submitted filename: Response to reviewers2.docx [file pone.0287667.s004.docx]

**Response to reviewer comments:**

Thank you for submitting your manuscript for review to PLoS ONE. After careful consideration, we feel that your manuscript will likely be suitable for publication if the authors revise it to address critical points raised previously  by the reviewer.   A major concern is still related to English language. At this time, we strongly suggest a professional copy-editing service.  Finally, it seems to be essential to include figures to represent study settings (there are free software that the authors can use to build their own figure) and also main data. For your guidance, a copy of the reviewers' comments was included below.

While revising your submission, please upload your figure files to the Preflight Analysis and Conversion Engine (PACE) digital diagnostic tool, <https://pacev2.apexcovantage.com/>. PACE helps ensure that figures meet PLOS requirements. To use PACE, you must first register as a user. Registration is free. Then, login and navigate to the UPLOAD tab, where you will find detailed instructions on how to use the tool. If you encounter any issues or have any questions when using PACE, please email PLOS at [figures@plos.org](mailto:figures@plos.org). Please note that Supporting Information files do not need this step.

**Response**:

We have got the manuscript copy edited for language by a professional editing agency (www.editage.com). We have enclosed the editing certificate from the agency.

We have created the map to show the geographical setting of the study using an open-source application (MAPWINDOW) and edited the base maps available from that source. We have added the reference to this software.

We have included a chart to report the data on malaria positivity and share of afebrile malaria in different rounds of screening.

We have used PACE to validate our figures. We have uploaded the valid tif files generated through PACE.
